# β-Phase Enhancement of Force Spun Composite Nanofibers for Sensing Applications

**DOI:** 10.3390/polym15173580

**Published:** 2023-08-29

**Authors:** Renato Wenceslao Aguirre-Corona, Karina Del Ángel-Sánchez, Nicolás Antonio Ulloa-Castillo, Juan José Rodríguez-Salinas, Daniel Olvera-Trejo, Imperio Anel Perales-Martínez, Oscar Martínez-Romero, Alex Elías-Zúñiga

**Affiliations:** 1 Institute of Advanced Materials for Sustainable Manufacturing, Tecnologico de Monterrey, Av. Eugenio Garza Sada Sur 2501, Monterrey 64849, N.L., Mexico; a00832503@itesm.mx (R.W.A.-C.); kdelangel@tec.mx (K.D.Á.-S.); daniel.olvera.trejo@tec.mx (D.O.-T.); anel.perales@tec.mx (I.A.P.-M.); 2 Center for Innovation in Digital Technologies, School of Engineering and Sciences, Tecnologico de Monterrey, Av. Eugenio Garza Sada Sur 2501, Monterrey 64849, N.L., Mexico; nicolas.ulloa@tec.mx; 3 School of Engineering and Sciences, Tecnologico de Monterrey, Av. Eugenio Garza Sada Sur 2501, Monterrey 64849, N.L., Mexico; juanjrdz@tec.mx

**Keywords:** polyvinylidene fluoride, barium titanate, graphene, piezoelectric, nanofibers, energy harvester, Forcespinning™

## Abstract

In this study, a piezoelectric harvesting device was developed using polyvinylidene fluoride (PVDF) nanofibers reinforced with either BaTiO_3_ nanoparticles or graphene powder. BaTiO_3_ nanoparticles were synthesized through the sol-gel method with an average size of approximately 32 nm. The PVDF nanofibers, along with the nanoparticle composites in an acetone-N,N-dimethylformamide mixture, were produced using a centrifugal Forcespinning™ machine, resulting in a heterogeneous arrangement of fiber meshes, with an average diameter of 1.6 μm. Experimental tests revealed that the electrical performance of the fabricated harvester reached a maximum value of 35.8 Voc, demonstrating the potential of BaTiO_3_/ PVDF-based piezoelectric devices for designing wearable applications such as body-sensing and energy-harvesting devices.

## 1. Introduction

Piezoelectric materials play a crucial role in electronic and medical applications due to their remarkable properties, including a high piezoelectric coefficient and the ability to efficiently generate clean energy. Among these materials, polyvinylidene fluoride (PVDF) stands out as a semi-crystalline polymer renowned for its chemical and thermal resistance, hydrolytic stability, and superior piezoelectric coefficient, making it an attractive choice for developing wearable electronics and sensors [1,2]. PVDF’s molecular structure consists of monomers (-CH_2_-CF_2_-)n, capable of forming four crystalline phases, α (monoclinic), β (orthorhombic), γ (monoclinic), and δ (monoclinic); the β phase exhibits the highest piezoelectric coefficient owing to its strong dipole moment [3,4,5,6,7,8,9]. However, PVDF naturally occurs in the non-polar α phase. Various techniques have been explored to enhance the piezoelectric coefficient of PVDF, such as incorporating nanofillers, mechanical stretching, chemical processes, thermal processes, and electrical polishing to increase the proportion of the desirable β phase [5,10].

The chemical process employed for enhancing the piezoelectric performance of PVDF-based sensors involves the use of solvents, with a preference for aprotic polar solvents [10,11]. Notably, N,N-Dimethylformamide (DMF), and acetone stand out as the most widely utilized solvents for nanofiber formation due to their ability to promote the β phase [12,13]. The combination of DMF with acetone proves particularly effective in enhancing the piezoelectric properties of PVDF. This mixture benefits from acetone’s capacity to facilitate a high evaporation rate during nanofiber formation, producing smooth nanofibers without bead formation [14,15]. Recent research indicates that a DMF-acetone ratio of 2:1 yields favorable results for the centrifugal force spinning process [10]. 

Reinforcing the PDVF nanofibers with nanofillers offers a promising avenue to enhance their properties and piezoelectric behavior [13]. Among the nanofillers, the perovskite-structured barium titanate (BaTiO_3_) emerges as a particularly attractive option for boosting piezoelectric sensor performance, owing to its high piezoelectric coefficient [16,17]. Notably, BaTiO_3_ has become a substitute for lead zirconate titanate (PZT), one of the best piezoelectric materials. BaTiO_3_ proves advantageous due to its biocompatibility, environmental safety, low cost, and widespread availability [1,7].

Another nanofiller that has improved the performance of PVDF-based harvesters is graphene, a two-dimensional structure with a high specific surface area. Graphene exhibits exceptional electrical conductivity, excellent thermal conductivity, and remarkable mechanical properties. Its potential as a constitutive material in sensors has been demonstrated, showcasing good energy conversion efficiency and high sensitivity [1,7,15,18]. A study conducted by Rahman et al. [19] explored the impact of adding graphene oxide (GO) and reducing it to reduced graphene oxide (RGO) through in situ thermal reduction on PVDF films created through solution castings. The results indicated a 51.3% increase in the output voltage for the PVDF/RGO nanocomposites at 0.1% wt.% in comparison to pure PVDF. This enhancement in piezoelectric properties was attributed to the conductive RGO nanofiller’s heterogeneous polarization and the specific interaction between the PVDF chain and the partially reduced graphene oxide functional group. Achaby et al. [20] created nanocomposite films using GO nanosheets and PVDF through solution casting. They discovered that a minimum concentration of 0.1 wt.% of GO was necessary to achieve only the β piezoelectric phase. At a lower concentration, the coexistence of both β and α phase was observed. While the inclusion of GO improved the tensile and thermal properties, the piezoelectric behavior was not examined. The enhancement of properties was attributed to the strong interfacial interaction between the macromolecular chains of PVDF and the surface of GO. Huang et al. [21] investigated the fabrication of flexible piezoelectric sensors by coating commercial fabrics with a composite material made of PVDF and graphene. These researchers discovered that the addition of graphene to PVDF resulted in an improvement in the β/γ electroactive phases. However, when the content of graphene exceeded 1 wt.%, the β/γ phases decreased. This was related to the interaction between graphene, n-methyl pyrrolidone, water, and PVDF during the experiment process. The sensors exhibited a sensitivity of 34 V N^−1^. Luo et al. [22] developed a tactile sensor by electrospinning a mat based on polyvinylidene fluoride-trifluoro ethylene (PVDF-TrFE) modified with BaTiO_3_ to serve as a piezoelectric layer, and organic electrodes of polyurethane (PU) nanofibers decorated with multi-walled carbon nanotubes. Compared with the reference material, piezoelectric performance increased by an impressive 187%, showcasing a linear relationship between the external force and the electrical output. Cho et al. [23] created a tactile sensor using a PVDF nanofiber membrane that was made through electrospinning. They used a mixture of DMF and acetone in a 2:1 volume ratio as solvents for the PVDF. Through a proof-of-concept experiment, they determined that static tactile sensing was successful. Even without physical contact, the piezoelectric voltage across the pair of Al electrodes showed a large piezoelectric response due to the continuous mechanical deformation of the PVDF membrane at a driving frequency of 25 Hz. Shi et al. [8] created piezoelectric nanogenerators using a combination of graphene, BaTiO_3_, and PVDF in nanocomposite fibers. During the electrospinning process, they used a mixture of solvents, DMF and acetone in a 2:3 ratio. The team found that including 0.15 wt.% graphene and 15 wt.% BaTiO_3_ in the piezoelectric device resulted in an output voltage of approximately 11 V and a maximum electric power of 4.1 μW when subjected to a loading frequency of 2 Hz and a strain of 4 mm.

According to the above, piezoelectric nanogenerators can be manufactured using various techniques, including spin coating [24], hot press [12], electrospinning [25], solvent casting [26], and deposition techniques [27,28]. Among these methods, electrospinning technology stands out as a widely used technology for fabricating electronic and biomedical devices, as well as for tissue engineering applications. Electrospinning proves to be more efficient and cost-effective than electrical poling, as it results in a higher β-phase fraction with permanent piezoelectric properties [18,29,30,31]. 

This article focuses on investigating the performance of a PVDF-piezoelectric nanogenerator doped with BaTiO_3_ and graphene as fillers. Forcespinning™ technology was chosen for producing the nanofibers due to its advantageous features over electrospinning, including compatibility with multiple conducting and non-conducting polymeric solutions and more important, high throughput [29,30,31]. Subsequently, the performance of the PVDF-based piezoelectric nanogenerator was examined using experimental data collected via a digital multimeter when the device was impacted by the Impact Testing Machine (Charpy) to quantitatively assess the energy generated. Thus, the results presented in this work focus on studying the performance of the piezoelectric materials in generating an electrical signal and their characterization.

## 2. Materials and Methods

### 2.1. Material

PVDF powder with a molecular weight of 534,000, N,N-dimethylformamide (DMF), titanium butoxide and barium acetate were all purchased from Sigma-Aldrich (St. Louis, MO, USA). Graphene nanopowder with 2–10 μm particle sizes was purchased from Nanostructured & Amorphous Materials, Inc. (Houston, TX, USA), and acetone was obtained from DEQ (Monterrey, NL, Mexico).

### 2.2. Synthesis of the BaTiO_3_ Nanoparticles

BaTiO_3_ nanoparticles were synthesized using the sol-gel method. Initially, 9.97 mmol of barium acetate (CH_3_COO)_2_ was mixed with 20 mL of acetic acid, 20 mL of distilled water and 5 mL of ethanol in a flask equipped with a reflux column to prevent solvent evaporation. The solution was stirred for 4 h to achieve a homogeneous solution. Next, titanium butoxide was added dropwise to the solution and stirred magnetically at 55 °C for 24 h. To promote gelation, 20 mL of distilled water was added to the solution and stirred for an additional 3 h. Then, the solution was dried in an oven at 90 °C for 24 h. Finally, the obtained material underwent thermal treatment at 900 °C for 3 h, resulting in the synthesis of BaTiO_3_ nanoparticles.

### 2.3. Fabrication of Piezoelectric Sensor

PVDF polymer powder (3.75 mg) was dissolved in a mixture of acetone (18.7 mL) and DMF (6.2 mL) in a 3:1 ratio and magnetically stirred at 85 °C for 1 h to obtain a polymer solution at 15 wt.%, which served as a polymeric matrix solution. The PVDF composite solutions were obtained by incorporating BaTiO_3_ and/or graphene nanofillers into the matrix solution and stirring for one hour at 65 °C to prevent particle agglomeration. The composite samples produced for this study are described in Table 1. The weight concentrations employed in this research were deliberately kept low to enhance the piezoelectricity of the developed PVDF-based materials [8].

The nanofiber meshes were produced using a centrifugal force spinning machine (Forcespinning™) equipped with a 27-gauge needle in the spinneret. The nanofibers were produced by spinning the machine to an angular speed of 5500 revolutions per minute (rpm) for a 1-min accumulation and were collected by using a metal crown positioned 25 cm away from the spinneret. Subsequently, the collected composite material was detached from the crown using a square metal frame with dimensions of 2.54 cm (about 1 in).

**Figure 1:**
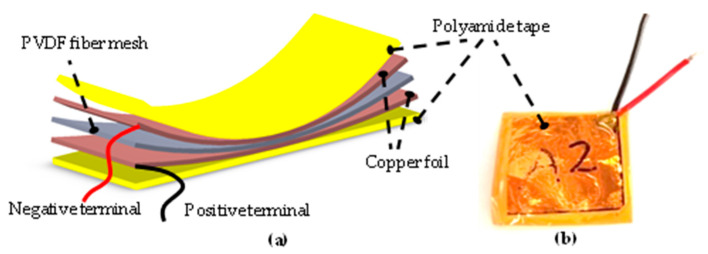
(**a**) Scheme and structure of the piezoelectric sensor; (**b**) optical photograph of an assembled device.

For the piezoelectric sensor architecture, the piezoelectric polymeric composite nanofibers were sandwiched between two copper foil tape electrodes (see Figure 1a). The device was covered with polyamide tape to protect the sensor from external contaminants (see Figure 1b).

## 3. Characterizations of the Synthesized BaTiO_3_ and Composite Nanofibers

The composite nanofibers’ morphology was studied using a scanning electron microscope (SEM) Zeiss EVO MA 25 (Oberkochen, Germany). Morphology and chemical composition were examined using secondary electrons (SE) and backscattered electrons (BSE). Energy-dispersive spectroscopic (EDS) analysis was also performed to obtain the elemental mapping images. The SEM micrographs were taken in three different areas, including the shores and the center of each sample, at a 5–10 kV voltage under high vacuum conditions. The size distribution of both BaTiO_3_ nanoparticles and composite nanofibers was determined using ImageJ software (FIJI it is public domain for open source software). 

Moreover, infrared analyses were conducted with an FTIR spectrometer (Perkin-Elmer Frontier, Waltham, MA, USA) using the Attenuated Total Reflectance (ATR) accessory. The samples were measured in the spectral range from 4000 to 400 cm^−1^, with a spectral resolution of 4 cm^−1^ and an average of 16 accumulated scans.

The crystal structure analysis of both the nanofiber meshes composite and the BaTiO_3_ nanoparticles was conducted using an X-ray diffractometer (XRD PanAnalytical, X’Pert Pro PW1800, Almelo, The Netherlands). Measurements were performed using Bragg-Brentano geometry in reflection mode, with a Cu K*α* radiation in the 2θ scanning range from 5° to 90° and a scan rate of 2°/min. The system was operated at 45 kV and 40 mA.

Thermal degradation analyses were carried out using a TGA instrument (Perkin-Elmer, Pyris 8000 New Castle, DE, USA). Measurements were carried out over a temperature range of 30 °C to 700 °C with a heating rate of 10 °C/min. Nitrogen (N_2_) gas was used as a purge gas (20 mL/min) in the temperature range from 30 °C to 600 °C, after which it was switched to oxygen (O_2_) for thermal oxidation.

**Figure 2:**
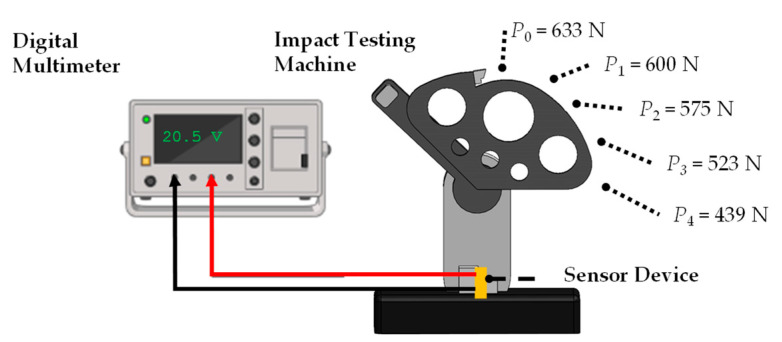
Schematic diagram of the experimental setup to test the piezoelectric response of the developed harvesters.

The piezoelectric behavior of the developed materials was investigated using a polymer impact testing machine (Charpy), Tinius Olsen model IT104 (Atlanta, GA, USA), as illustrated in Figure 2. To measure the piezoelectric response, a portable digital multimeter, Agilent U1251A (Santa Clara, CA, USA)(using hold max feature), was connected to register the maximum output voltage produced by the sensor device when subject to the mechanical force that resulted from the impact tool. The typical voltage signal generated by the piezoelectric sensor was captured with an oscilloscope, as depicted in Figure S1. Additionally, consistent maximum voltage measurements using a digital multimeter are also presented in Figure S2 and detailed in the Supplementary Materials.

The piezoelectric tests were conducted from five distinct starting positions of the impact tool, as depicted in Figure 2. Commencing with the first impact at the maximum height of the impact tool (P_0_), this position aligns with the plane oriented 0° from the sensor surface. Subsequent measurements were taken every 15 degrees clockwise from P_0_. The resulting impact forces were 633 N for P_0_, followed by forces of 600, 575, 523 and 439 N for P_1_, P_2_, P_3,_ and P_4_, respectively. These forces correspond to impact energy values of 0.282, 0.249, 0.231, 0.188 and 0.160 kilogram force-meter, respectively. The force measurements were captured using an impact hammer, specifically the Kistler model 9722A500 (Amherrst, NY, USA). 

To calculate the piezoelectric coefficient (*d*_33_), the average of the maximum voltage output from five measurements was taken using a rectifier circuit. The circuit was connected to a capacitor to allow the electrical signals to pass through, generating a conditioned signal. The mathematical expression to determine the piezoelectric coefficient value is given as:

(1) $$d_{33}=\frac{Q}{F}=\frac{C_{m}V_{m}}{F}$$

where *Q* is the electric charge, which can be calculated from the capacitance (0.01 μF connected in parallel to the sensor) multiplied by the maximum voltage produced by the device, and *F* is the maximum force exerted. The sensitivity associated with the piezoelectric response was calculated using the following equation:

(2) $$Sensitivity=\frac{V}{F}$$

where *V* is the mean maximum voltage produced by the device, and *F’* is the force applied to generate that voltage [7].

## 4. Results and Discussions

### 4.1. Morphology and EDS Elemental Mapping

**Figure 3:**
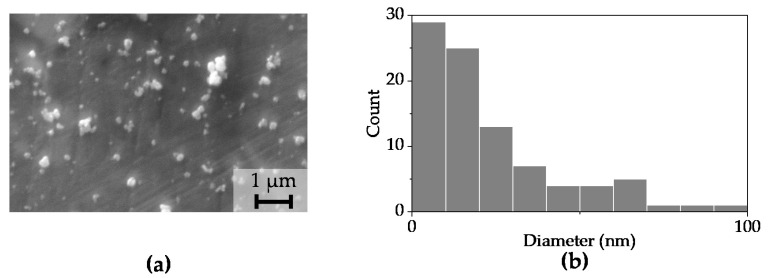
(**a**) SEM image of BaTiO_3_ nanoparticles; (**b**) particle size distribution. Upon examination of the micrograph, it was observed that agglomerates of nanoparticles were present.

Figure 3a displays an SEM micrograph capturing the powder of BaTiO_3_ nanoparticles that were obtained via the sol-gel method, while Figure 3b shows the particle size distribution, indicating an average size of 32 nm. 

**Figure 4:**
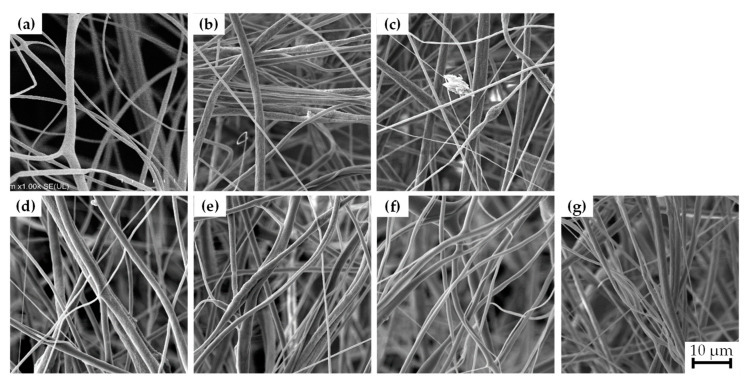
SEM micrographs of nanofiber meshes for each composite shows diameters without alignment and different arrangement in all samples of PVDF and mixes of PVDF with BaTiO_3_ and graphene fiber meshes. (**a**) PVDF fibers; (**b**) BaTiO_3_15%/PVDF fiber; (**c**) G0.05%/PVDF fiber; (**d**) BaTiO_3_15%/G0.05%/PVDF fiber; (**e**) BaTiO_3_5%/PVDF fiber; (**f**) G0.15%/PVDF fiber; (**g**) BaTiO_3_5%/G0.15%/PVDF fiber.

Figure 4 illustrates the morphology analysis of the PVDF composite meshes (A, B, C, D, E, F, and G). The PVDF nanofibers (sample A) exhibited a smooth structure without material agglomerations, commonly seen as beads along the nanofibers, with a mean diameter of 1.6 µm. On the other hand, PVDF nanofibers reinforced with BaTiO_3_ and/or graphene (samples B, C, D, E, F, and G) exhibited beads, primarily attributed to particle agglomeration due to the higher density of BaTiO_3_ nanoparticles compared to PVDF powder, resulting in some agglomerates of BaTiO_3_. This finding agrees with the results obtained by Hoejin et al. [32].

The measured diameters sizes presented in Table 2 for the produced composite nanofibers were smaller than those of the reference PVDF nanofibers (sample A), ranging from 1 µm to 1.4 µm, which agrees with the findings reported in [33,34] for similar systems. The variance in diameter of the composite nanofibers can be attributed to the incorporation of the nanofiller, which, combined with the rapid solvent evaporation ratio during the fiber formation, modifies the viscosity of the solution leading to nanofiller agglomerations and bead formation.

**Figure 5:**
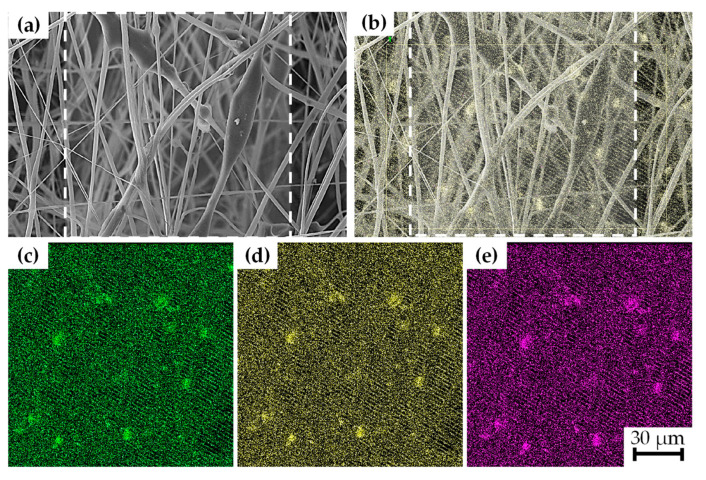
(**a**) SEM image of the nanofibers with BaTiO_3_ nanoparticles; (**b**) composition of BaTiO_3_ nanoparticles and nanofiber meshes obtained by backscattering SEM, EDS showing mass distribution for (**c**) Ti, (**d**) O, (**e**) Ba.

The chemical composition of the samples was investigated using energy dispersive spectroscopy (EDS). Figure 5 displays EDS analysis for sample G, revealing mappings for titanium (Ti), oxygen (O), and barium (Ba). The result indicated a homogeneous distribution of BaTiO_3_ within the nanofibers; however, some particle agglomeration was observed with average sizes of 6 µm.

### 4.2. XRD Measurements

**Figure 6:**
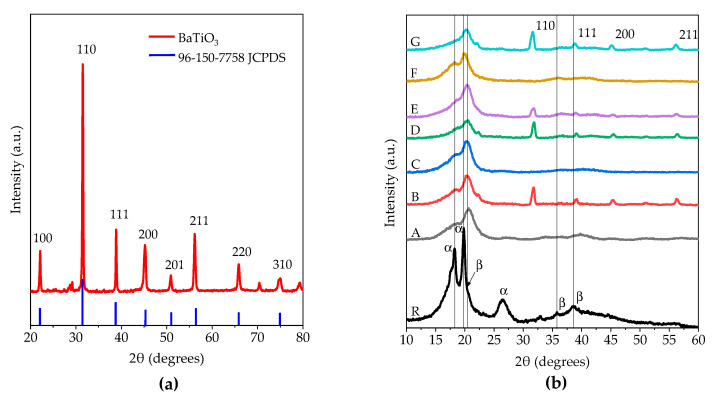
XRD diffractograms from (**a**) crystalline phase of BaTiO_3_ and (**b**) piezoelectric composits and their reference materials, where sample R shows PVDF polymeric powder planes of α and β phases’ coexistence, and sample A to sample G show the presence of β phase at 2θ = 20.5°, principally, in all samples.

The XRD diffractograms for the synthesized BaTiO_3_ nanoparticles are shown in Figure 6a. As evident from Figure 6a, the characteristic crystallographic planes of a BaTiO_3_ with cubic cell and a space group Pm-3m are presented according to the JCPDS crystallographic card of BaTiO_3_ (96-150-7758). The positions of the main crystallographic planes are located at 31.5°, 38.9°, 45.3°, and 56.1° in 2θ for the planes (110), (111), (200), and (211), respectively.

Figure 6b shows the analyzed samples of the reference PVDF powder and samples A, B, C, D, E, F, and G with their corresponding nanofillers at different concentrations, as listed in Table 1. Diffractograms depict the coexistence of α and β phases. α phase is exhibited by the reflections located at 2θ = 18.3°, 19.8°, and 26.6°, while the β phase appears at 2θ = 20.5°, 35.7° and 38.9°, respectively. PVDF fibers only show a broad peak at 2θ = 20.5° corresponding to the β phase, along with faint signs at 2θ = 18.3° and 19.8° which are related to the α phase. Upon incorporating BaTiO_3_ nanoparticles and graphene, the positions of peaks attributed to β phase are not modified. However, the main crystallographic planes from BaTiO_3_ are presented at 31.5°, 38.9°, 45.3°, and 56.1° in 2θ only for those samples that contain BaTiO_3_ as filler. On the contrary, for those samples that include only graphene (samples C and F) as filler, a small shoulder due to the α phase is located at 18.3° in 2θ. Wu et al. reported that peaks at 2θ = 18.2° and 26.5° can be attributed to the α phase, and those at 2θ = 20.2° to the β phase [35]. Regarding graphene addition, it was impossible to determine any signal for this due to the main crystallographic planes overlapping with those of the β phase [36]. The XRD analysis results confirmed that different mass concentrations of fillers as dopant material used in the PVDF polymer matrix alter the composition and properties of PVDF. 

### 4.3. Fourier-Transform Infrared Spectroscopy Measurements

**Figure 7:**
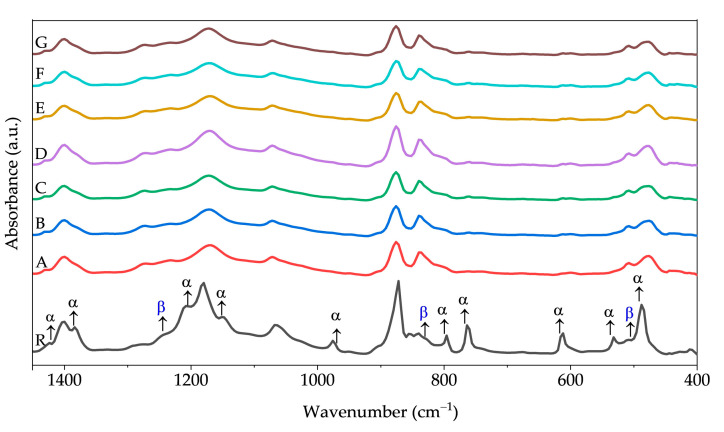
FTIR spectra for the composite materials manufactured by Forcespinning™. The PVDF powder shows that the α and β phases coexist, while in the fiber meshes the β phase increases.

The FTIR technique was used to investigate the absorption bands in the fiber meshes. For the composite samples, FTIR measurements revealed the presence of both α and β phases, with an increase in the intensity of the peaks belonging to the β phase compared to the raw material. Figure 7 and Table 3 illustrate the absorption bands associated with each phase. The spectrum of the PVDF reference powder depicts the characteristic absorbance bands corresponding to α phase at 489 cm^−1^ (CF_2_ waging), 533 cm^−1^ (CF_2_ bending), 615 and 764 cm^−1^ (CF_2_ bending and skeletal bending), and 795 and 975 cm^−1^ (CH_2_ rocking), according to Thakur et al. [37], and at 1149 and 1382 cm^−1^ [38]. Bands of β phase are located at 510, 840 cm^−1^ (attributed to CH_2_ rocking, CF_2_ stretching and skeletal C-C stretching), and 1276 cm^−1^ [27,37]. Notably, the β phase intensity increases with the addition of a nanofiller to the solution compared to the PVDF powder. Table 4 displays the β phase percentage presented in all samples reported in this study, which was calculated utilizing the Lambert–Beer equation. It is evident from Table 4 that the percentage of β phase decreases in the material samples containing graphene. Notice from Table 4 that the maximum β phase content of 83% was calculated for sample G. The presence of the bands corresponding to the β phase can be correlated with the results obtained from the XRD analysis. 

### 4.4. Thermogravimetric Analysis

**Figure 8:**
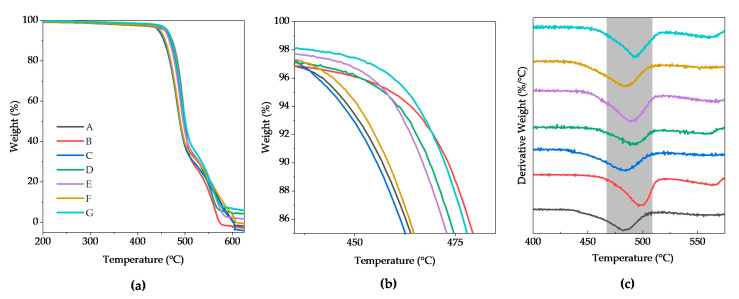
(**a**) TGA curves of PVDF with different concentrations of BaTiO_3_ and graphene, (**b**) zoom of TGA curves that shows the 10% weight loss temperature, and (**c**) derivative thermogravimetric (DTG) curves.

First, during the thermal degradation of PVDF at low temperatures, the carbon skeleton structure and the carbon–hydrogen bonds of PVDF are removed. Botelho et. al., [39] observed that this process involves the scission of carbon-hydrogen bonds primarily due to their lower bond strength (between 410 and 460 kJ/mol) compared with C–F. Decomposition temperature at 10% of weight loss (T_10%_) was increased for the composite materials compared to PVDF fibers from 456.8 °C to 473.1 °C (see Figure 8a,b, and Table 4). This thermal stability increase was evidenced only for those materials reinforced with BaTiO_3_ and a combination of BaTiO_3_ and graphene; however, this increment was not presented for those materials reinforced only with graphene. During the subsequent step, the maximum decomposition temperature (T_p_) occured at around 500 °C (see Figure 8c), and this behavior was similar to T_10%_ since the T_p_ for the piezoelectric composite materials improved compared to the reference material evidencing the thermal stability. The T_p_ incremented from 482.6 °C for the PVDF fibers to a maximum value of 498.8 °C for the BaTiO_3_15%/PVDF fibers.

### 4.5. Piezoelectric Tests

The output voltage values obtained for the developed PVDF-based nanogenerators are summarized in Table 5. Interestingly, while sample G exhibited the highest percentage of the β phase, its performance was comparatively low, generating a maximum of only 14.5 V. Nevertheless, sample B, which β phase content is only 81.4% according to FTIR results, exhibited the highest voltage output (35.8 V), resulting in a 70% more than the PVDF fiber reference material. Since sample G contains 5 wt.% of BaTiO_3_ and 0.15 wt.% of graphene, and sample B only contains 5 wt.% of BaTiO_3,_ further investigation into different graphene concentrations is necessary to identify the optimal performance-enhancing configuration. Notably, Shi and colleagues [8] showed how important the interaction between BaTiO_3_ and functionalized graphene is. Their findings were consistent with previous research [7,15,40,41,42]. Notice that the materials reinforced with BaTiO_3_ outperformed those reinforced with graphene, as is evident from the improved sensitivity and *d*_33_ values.

The piezoelectric coefficient measurement utilized an electrical circuit with a rectifier bridge, where the piezoelectric device was connected to enable the passage of the electrical signals to a capacitor, generating a continuous signal. In order to evaluate the piezoelectric device’s behavior, various forces were applied to the sensor that showed the highest level of performance. Various impact positions were employed to induce differences in the voltage generation (as shown in Figure 2). 

**Figure 9:**
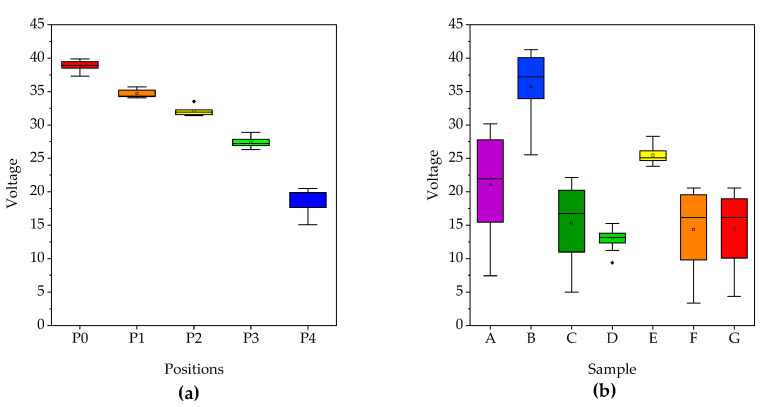
(**a**) Maximum output voltage values recorded from sample B for the five starting impact tool positions (different forces). (**b**) Maximum voltage values obtained for all piezoelectric samples.

Notably, the voltage produced was directly proportional to the force exerted; the lower the force applied, the lower the voltage generated. The behavior is visually illustrated in Figure 9, showcasing the results for different starting positions of the impact tool, namely, P_0_, P_1_, P_2_, P_3_, and P_4_ (see Figure 9a). The impact signal was generated when the Impact Testing Machine impacted the sensor device. The physical phenomenon is observed when mechanical stress is applied to the PVDF fiber arrangement. Figure 9b shows the voltage generated by all tested samples with P_0_ = 633 N, and the BaTiO_3_ and graphene nanofillers. According to the results of piezoelectric coefficient (*d*_33_) shown in Table 5, it can be observed a higher behavior for the device that contains BaTiO_3_ at 15 wt.% compared with the PVDF fiber reference, showing an increment of 21%. Conversely, when BaTiO_3_ is 5 wt.%, the performance decreases by 14%. Piezoelectric coefficients were computed using the average of the maximum voltage output from five measurements while using a rectifier circuit, which are 0.99, 1.20 and 0.85 V for samples A, B and E, respectively. An oscilloscope and multimeter were used to verify the Supplementary Material’s results illustrated in Figures S1a and S2c.

Comparing our obtained piezoelectric coefficient with other studies, we found noteworthy results. For example, Zhang et al. achieved a maximum *d*_33_ value of 39.73 pC/N with graphene concentration of 0.11 vol%, and an 80.5% increase compared to pure PVDF [43]. Yaqoob et al. designed tri-layer piezoelectric harvesters (PVDF-BTO/nGr/PVDF-BTO), with their PNG configuration delivering the highest output voltages (10 Vpp) and currents (2.5 μApp), and 2 Vpp from human finger tapping [36]. While they do not report the piezoelectric coefficient, they underscore the diverse performance achievements in various setups. Rahman et al., [19] reported an increment of 51.38% in the output voltage for the piezoelectric devices based on PVDF/RGO compared to pure PVDF. The authors do not report the use of an electrical rectifier circuit and the piezoelectric coefficient. Calió et al. highlighted the importance of *d*_33_ as a widely used mode for piezoelectric-based devices, as evidenced in various configurations, including cantilever tests [44].

The results presented in this work only apply to sensing and monitoring applications where a change in pressure is required to produce a voltage signal that subsequently can be amplified to be used as an analog for the sensing process or discrete for On–Off operations.

The use of BaTiO_3_/PVDF composites has demonstrated to have a great potencial in the field of electric power generation, specifically in the nanogenerators field as reported in the works of Zhang [45] and Qin [46], respectively. These applications consider the same principle considered in this work, as electrical energy can be obtained with an application of mechanical force.

## 5. Conclusions

In this study, we successfully developed piezoelectric nanofiber meshes by incorporating both BaTiO_3_ nanoparticles, obtained via the sol-gel method with an average particle size of around 32 nm, and commercial graphene, as fillers in a PVDF polymeric matrix using the Forcespinning™ technique. The nanofibers containing BaTiO_3_ particles exhibited superior performance compared to those with graphene, making them promising candidates for piezoelectric devices.

SEM micrographs confirmed the random distribution of nanofibers with an average diameter of approximately 1.0 μm, and EDS elemental mappings corroborated a homogenous distribution of BaTiO_3_ nanofiller in the fiber meshes. FTIR spectroscopy revealed noticeable changes in the spectra, particularly with more intense peaks observed in the bands associated with the β phase, indicating a higher concentration of the β phase in the composite nanofibers (~80%) compared to the raw material (~36%), and XRD analysis validated the existence of β phase planes in the fiber meshes, along with the characteristic planes of the perovskite-type structure of BaTiO_3_. Thermal degradation analysis further confirmed that the thermal stability was increased due to the presence of BaTiO_3_ and graphene as fillers.

Piezoelectric testing using an impact tester demonstrated the generation of a maximum voltage of 35.8 Volts in samples with BaTiO_3_ nanoparticles (samples B and E). In contrast, samples containing graphene (C, D, F, and G) showed lower performance. The results emphasized the significant influence of the addition of nanofillers on achieving higher β phase content, ultimately contributing to the successful fabrication of piezoelectric devices capable of functioning as sensors or nanogenerators, with a wide range of potential applications.

Given the research applications shown in the literature and the encouraging results presented in this study, it is highly feasible to explore the implementation of these nanofiber meshes in nanogenerator applications. The need to continue this research in the proposed new direction with the commitment to dedicating future efforts and resources to advance this field of study is recognized.

## Figures and Tables

**Table 1:** Composition of the composite solutions used to develop the composite nanofibers by centrifugal force spinning.

PVDF Composite Samples	Description	PVDF (wt.%)	BaTiO_3_ (wt.%)	Graphene (wt.%)
R	PVDF powder			
A	PVDF fiber	15%	-	-
B	BaTiO_3_/PVDF	15%	15%	-
C	Graphene/PVDF	15%	-	0.05%
D	BaTiO_3_/Graphene/PVDF	15%	15%	0.05%
E	BaTiO_3_/PVDF	15%	5%	-
F	Graphene/PVDF	15%	-	0.15%
G	BaTiO_3_/Graphene/PVDF	15%	5%	0.15%

**Table 2:** Average diameter of nanofibers mesh manufactured.

Key	Solution	Diameter Value (μm)
A	PVDF	1.6
B	BaTiO_3_/PVDF	1.4
C	Graphene /PVDF	1.4
D	BaTiO_3_/Graphene/PVDF	1.1
E	BaTiO_3_/PVDF	1.0
F	Graphene/PVDF	1.2
G	BaTiO_3_/Graphene/PVDF	1.2

**Table 3:** Wavenumber position of α and β phases in FTIR spectra with their functional group and vibrational mode.

Phase	Wavenumber (cm^−1^)	Functional Group	Vibrational Mode
α	489	CF_2_	Waging
β	510	CF_2_	Bending
α	533	CF_2_	Bending
α	615	CF_2_	Bending
α	764	CF-CH-CF	Skeletal bending
α	795	CH_2_	Rocking
β	840	CH_2_, CF_2_, C-C	Rocking, stretching, skeletal stretching
α	975	CH_2_	Rocking
α	1149	CF	Stretching
β	1276	---	---
α	1382	---	---

**Table 4:** β phase percentage in all samples worked on this paper and parameters of thermal stability obtained from TGA and DTG curves. A_α_ is the value of the characteristic α phase wavelength at 764 cm^−1^, A_β_ is the value of the characteristic β phase wavelength at 840 cm^−1^, T_10%_ corresponds to the decomposition temperature at 10% of weight loss, and T_p_ is the maximum decomposition temperature.

Samples	A_α_	A_β_	β	β Percentage	T_10%_ (°C)	T_p_ (°C)
R	0.1676	0.119	0.36	36.0%	-	-
A	0.0233	0.1371	0.823	82.3%	456.8	482.6
B	0.0225	0.1242	0.814	81.4%	473.1	498.8
C	0.026	0.1206	0.786	78.6%	455.5	483.3
D	0.0303	0.1652	0.812	81.2%	468.5	492.9
E	0.0195	0.1106	0.818	81.8%	466.8	490.5
F	0.0219	0.1062	0.793	79.3%	458.2	483.9
G	0.0191	0.1176	0.83	83.0%	472.3	492.4

**Table 5:** Voltage, piezoelectric sensitivity, and piezoelectric coefficient (*d*_33_) values for each produced PVDF-based nanogenerator device.

Sample	A	B	C	D	E	F	G
Maximum OutputVoltage (V)	21.0	35.8	15.3	13.0	25.5	14.4	14.5
Sensitivity (V/N)	7.6	12.91	5.5	4.7	9.2	5.2	5.2
*d*_33_ (pC/N)	16.0	19.4			13.8		

## Data Availability

Not applicable.

## Supplementary Materials

The following supporting information can be downloaded at: https://www.mdpi.com/article/10.3390/polym15173580/s1. Figure S1: (a) Oscilloscope screen capture displaying the recorded voltage measurement during the impact on Sample A, (b) Corresponding replica; Figure S2: Frames taken from recorded video of a typical measurement using a digital multimeter Agilent U1251A, before (a), during (b) and after (c) impacting the device of material A.
