# Supplementary material for: β-Phase Enhancement of Force Spun Composite Nanofibers for Sensing Applications
Source: Polymers (Basel). 2023 Aug 29;15(17):3580. doi: 10.3390/polym15173580 (PMC10490387; doi:10.3390/polym15173580)
Supplement: Supplementary file 1 [file polymers-15-03580-s001.zip › polymers-2518231-supplementary.pdf]

polymers
MDPI

Supplementary Materials

β-Phase Enhancement of Force Spun Composite Nanofibers for Sensing Applications

Renato Wenceslao Aguirre-Corona [1], Karina Del Ángel-Sánchez [1], Nicolás Antonio Ulloa-Castillo [2], Juan José Rodríguez-Salinas [3], Daniel Olvera-Trejo [1], Imperio Anel Perales-Martínez [1], Oscar Martínez-Romero [1,*] and Alex Elías-Zúñiga [1,*]

1 Tecnologico de Monterrey, Institute of Advanced Materials for Sustainable Manufacturing, Av. Eugenio Garza Sada Sur 2501, Monterrey, N.L., México, 64849. a00832503@itesm.mx (R.W.A.-C.); kdelangel@tec.mx (K.D.Á.-S.); daniel.olvera.trejo@tec.mx (D.O.-T.); anel.perales@tec.mx (I.A.P.-M.)
2 Tecnologico de Monterrey, Center for Innovation in Digital Technologies, School of Engineering and Sciences, Av. Eugenio Garza Sada Sur 2501, Monterrey, N.L., México, 64849. nicolas.ulloa@tec.mx
3 Tecnologico de Monterrey, School of Engineering and Sciences, Av. Eugenio Garza Sada Sur 2501, Monterrey, N.L., México, 64849. juanjrdz@tec.mx
* Correspondence: oscar.martinez@tec.mx (O.M.-R.); aelias@tec.mx (A.E.-Z.); Tel.: +52-81-83-582000 (ext. 5653) (A.E.-Z.)

Experimental Voltage Measurements

Voltage measurements of the piezoelectric devices were conducted using a benchtop oscilloscope, specifically the Tektronix model TSD 2014B. This oscilloscope was connected to a computer to record data in CSV files and to capture the voltage signal generated during impact testing with the Charpy device. The oscilloscope settings were adjusted to display a signal with a maximum peak of 30V (corresponding to a 5V vertical tick), and the trigger level was set at 400mV to initiate and maintain the measurement.

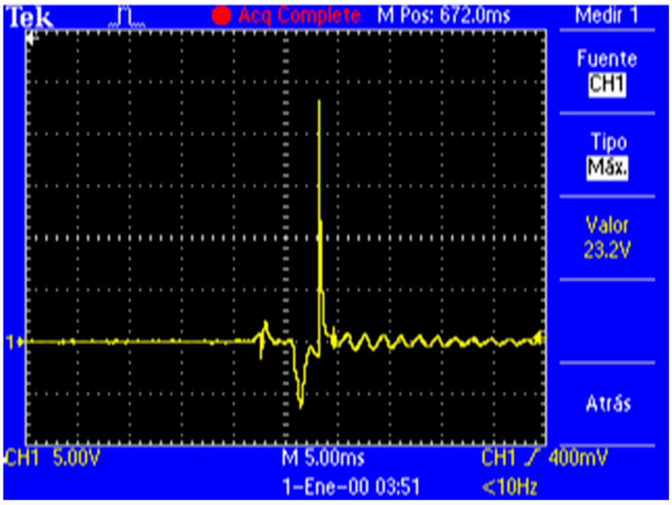

(a)

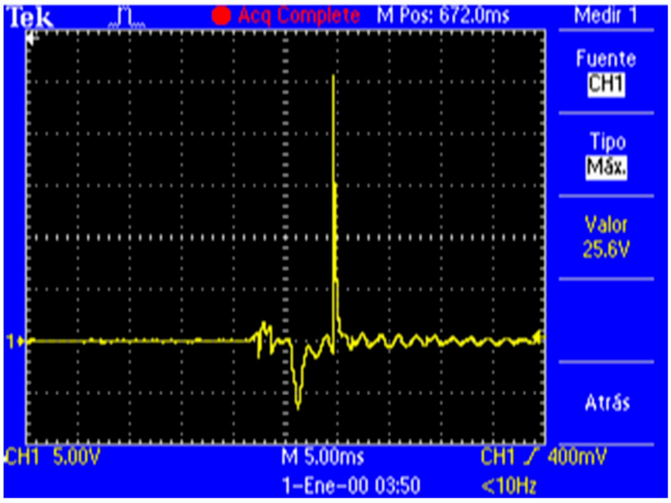

(b)

Figure S1. (a) Oscilloscope screen capture displaying the recorded voltage measurement during the impact on Sample A, **(b)** Corresponding replica.

Figure S1a and its corresponding replica S1b clearly illustrate that the impact results in a minor negative peak of -6.1V. Simultaneously, the oscilloscope's measuring features registered maximum peaks of 23.2V and 25.6V. This positive peak duration spans around

1ms. Since the sampling frequency of 50kHz, it is anticipated that there may be some error in detecting the exact maximum voltage. In essence, the 20μs sampling interval cannot consistently capture the same peak value. For this reason, 5 replicas were averaged.

The measurement was further confirmed using a digital multimeter, Agilent model U125A. Although a standard digital multimeter might not be the optimal tool for capturing rapid or transient voltage signals, as generated by the piezoelectric device during an impact test, this multimeter offers a 'hold maximum' feature that reliably produces consistent peak values. A representative measurement was captured on video, and frames extracted from the video are presented in Figure S2, depicting the moments before, during, and after the impact.

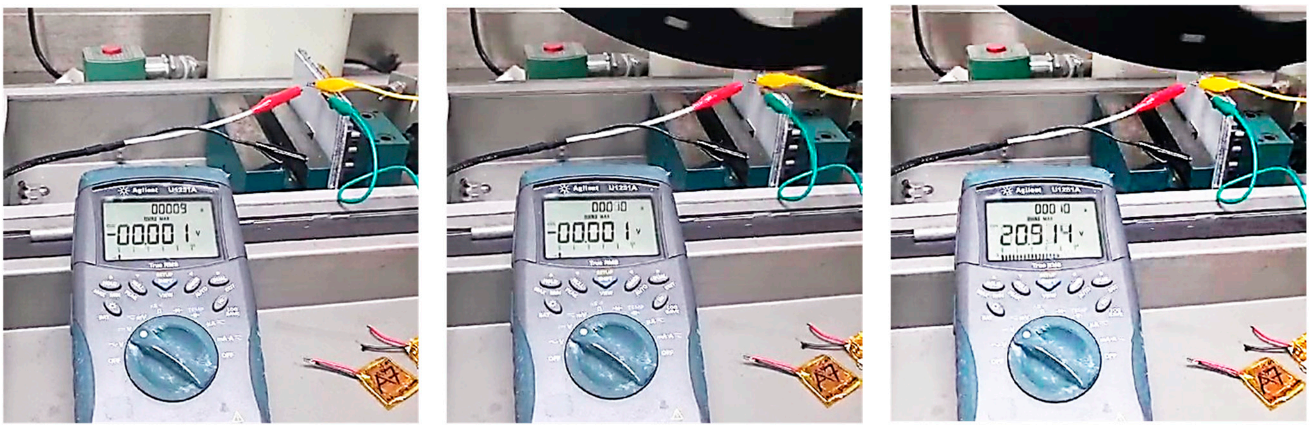

(a) **(b)** **(c)**

Figure S2. Frames taken from recorded video of a typical measurement using a digital multimeter Agilent U1251A, before (**a**), during (**b**) and after **(c)** impacting the device of material A.

The video in Figure S2 corresponds to an impact test on Sample A, resulting in a consistent maximum peak of 20.9 V as measured by the digital multimeter. Consequently, the majority of measurements were obtained using the digital multimeter.
